# Homœopathic Medical College of Missouri

**Published:** 1895-04

**Authors:** 


### HOMŒOPATHIC MEDICAL COLLEGE OF MISSOURI.

### The Thirty-Sixth Commencement and Banquet at St. Louis.

The Thirty-sixth Annual Commencement exercises of the Homœopathic Medical College of Missouri were held on the evening of March 21st, at the Pickwick Theatre, Dr. Wm. C. Richardson, Dean, presiding. Degrees were conferred by Dr. W. A. Edmonds, President of the Board of Trustees, upon the following: Mary Elliott Beall, George Brickbauer, Peter Brickbauer, T. L. Carriere, Emile J. Chalfant, W. C. Dieterichs, G. A. Mellies, C. E. Ross, E. R. Schoen, E. H. Tincher. Prizes for exceptional excellence were also awarded to G. Brickbauer in Materia Medica; to P. Brickbauer in Obstetrics, and to G. A. Mellies in Gynæcology. The Faculty prize was awarded to P. Brickbauer, and the position of Resident Physician given to C. E. Ross at the Children's Hospital and to G. A. Mellies at the Good Samaritan Hospital.

The report of Dean Dr. Wm. C. Richardson was very encouraging, and showed the largest enrollment in the Freshman Class ever made since the organization of the college. The principal address was that of the Rev. Geo. C. Adams, who spoke in behalf of the Faculty. The programme was interspersed with musical selections rendered by professional talent, and the whole affair was most creditable to the college.

THE ALUMNI BANQUET.

The Alumni of the Homœopathic Medical College of Missouri held a banquet on the evening of March 20th, in the Ladies Ordinary of the Southern Hotel. About one hundred and twenty-five persons were present, one-fifth of that number being ladies. The banquet halls and tables were handsomely decorated with plants and flowers, and sweet strains of music were rendered by an orchestra.

After an elaborate *menu* had been served Dr. I. D. Foulon made an interesting address on Hahnemann, the founder of the Homœopathic School of Medicine. The subjects of other toasts were "The College," "The Doctor, Then and Now," "The Alumni Association," "The Family Physician," "Homœopathy," and the "Brand New Doctor," which were responded to by L. C. McElwee, T. Griswold Comstock, Mary U. Sargent, Rev. Frank G. Tyrrell, J. Martine Kershaw, and P. Brickbauer. The affair was most enjoyable, and it was a late hour when the company dispersed.

